# Supplementary figures and images for: Effect of 17β-estradiol on the daily pattern of ACE2, ADAM17, TMPRSS2 and estradiol receptor transcription in the lungs and colon of male rats
Source: PLoS One. 2022 Jun 28;17(6):e0270609. doi: 10.1371/journal.pone.0270609 (PMC9239479; doi:10.1371/journal.pone.0270609)

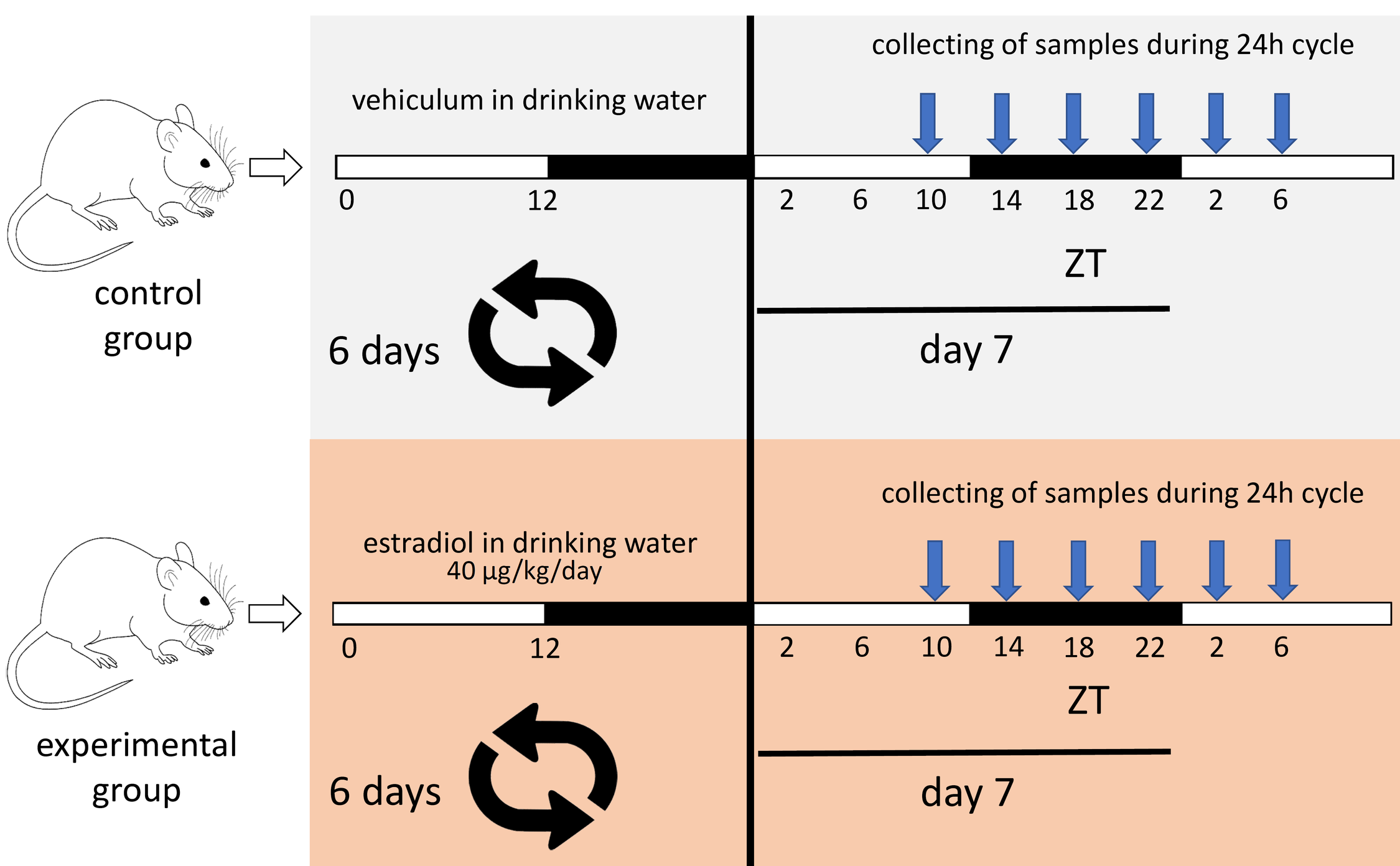

Supplement: S1 Fig — Adult male Wistar rats were synchronized to a light/dark (L, white rectangle; D, black rectangle; respectively) cycle with lights on at 7 a.m. 17β-estradiol (E2) was administered to rats (n = 25) in drinking water in a concentration of 40 μg/kg/day for 7 days. Animals from the control group (n = 25) received the E2 vehicle. Tissue sampling began on day 7 after the initiation of E2 treatment during a whole 24-h cycle, with the first time point at Zeitgeber time 10. ZT0 is defined as the beginning of the light phase, and ZT12 corresponds to the beginning of the dark phase of the LD cycle. Samples were taken at 4-h intervals at ZT10, ZT14, ZT18, ZT22, ZT2 and ZT6. At each time point, 4–5 controls and the same number of E2-treated rats were used. (TIF) [file pone.0270609.s001.tif]

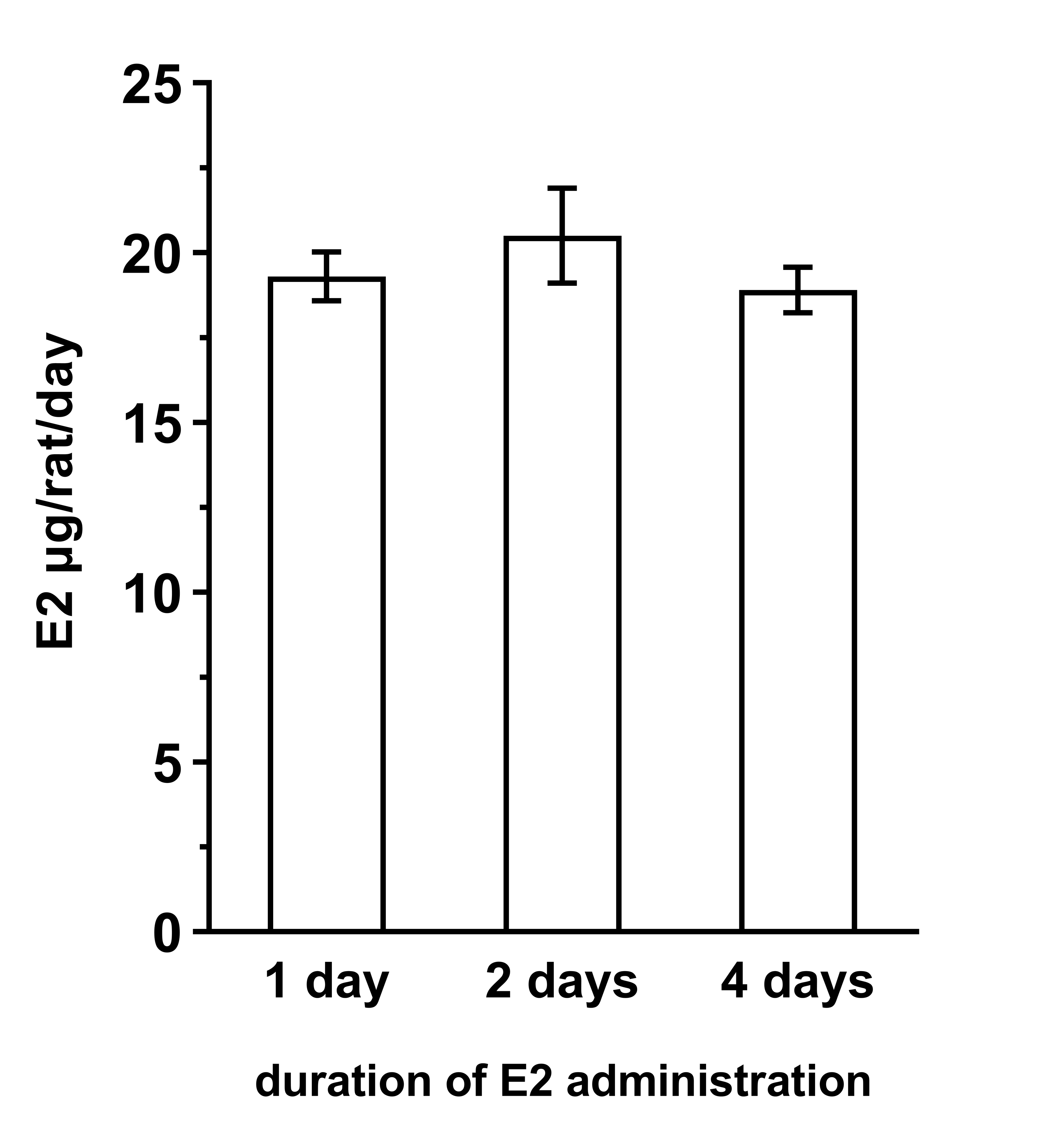

Supplement: S2 Fig — n = 25, measured E2 concentration in water provided to control is undistinguishable from zero (less than 7.5 pg/rat/day). The x-axis shows time course of experiment. (TIF) [file pone.0270609.s002.tif]

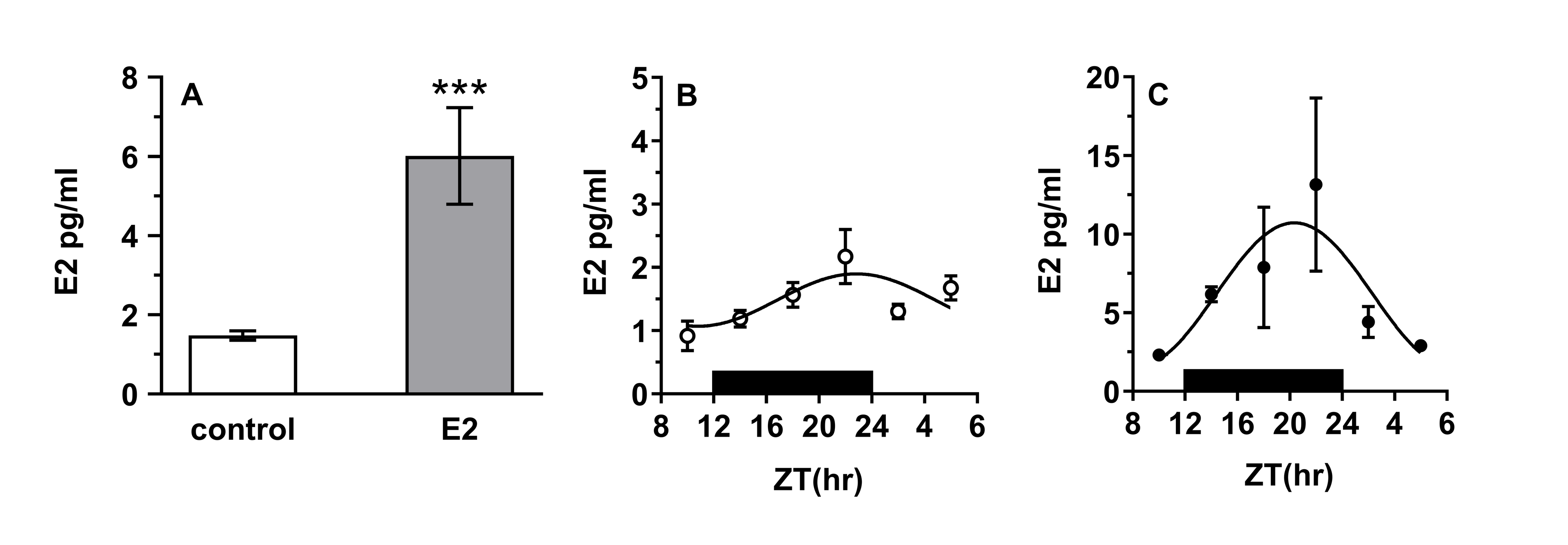

Supplement: S3 Fig — Averaged E2 plasma levels in control (white column, n = 25) and E2 treated (grey column, n = 25) rats (A). Daily profile in E2 levels in plasma of control (B, white circles) and E2 treated rats (C, black circles). In some cases SEM is indistinguishable from symbol. Solid line shows significant Cosinor fit (P < 0.05). Black rectangle on x-axis corresponds to dark phase of LD cycle. *** P < 0.001, unpaired t-test. (TIF) [file pone.0270609.s003.tif]

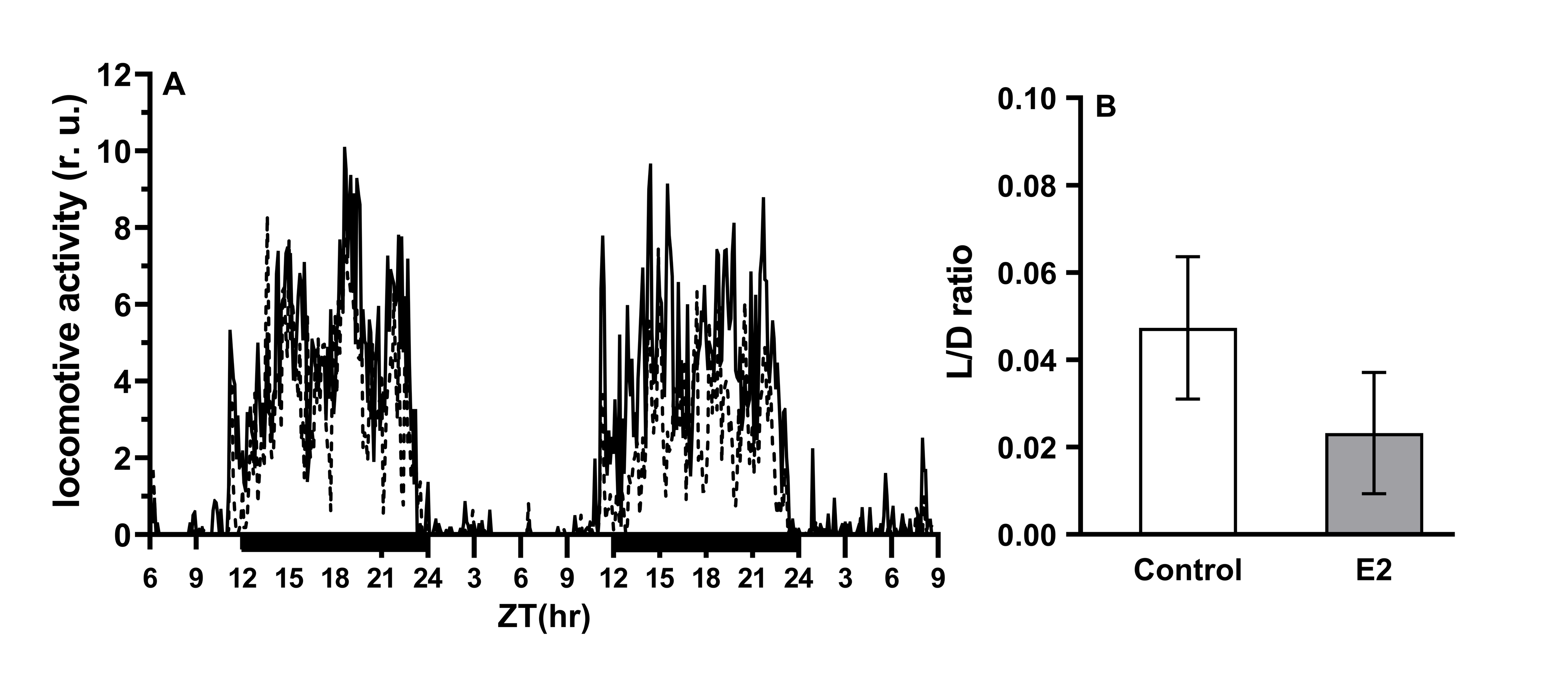

Supplement: S4 Fig — (A). The black bar at the bottom of the graph represents the dark phase of the LD cycle. (B) L/D is the ratio of activity measured during the light and dark phases of the LD cycle. (TIF) [file pone.0270609.s004.tif]

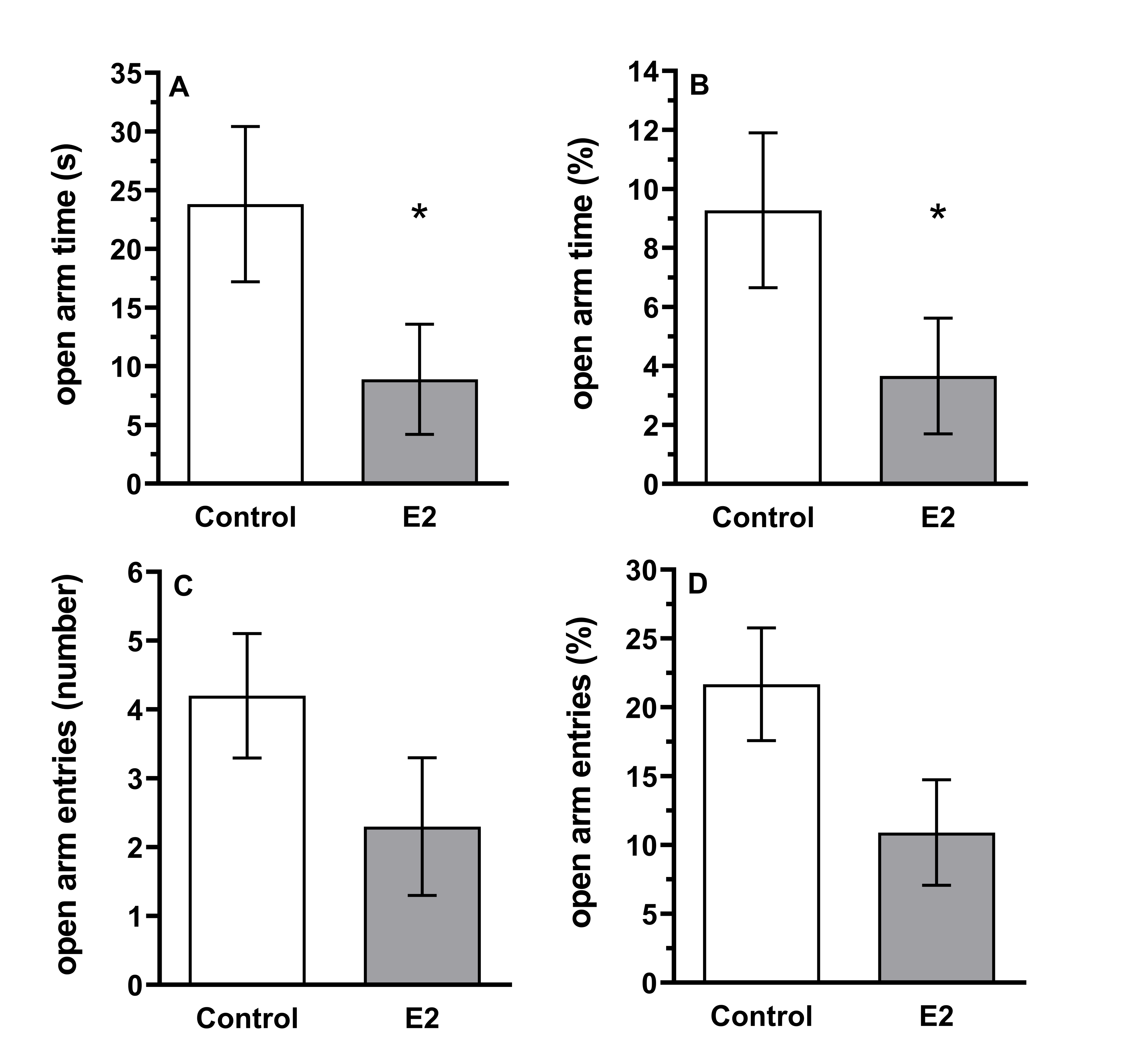

Supplement: S5 Fig — Number of entries and time spent in the open arms of the elevated plus-maze, expressed in absolute and relative units. E2–17β-estradiol, * P < 0.05, Mann-Whitey test. (TIF) [file pone.0270609.s005.tif]

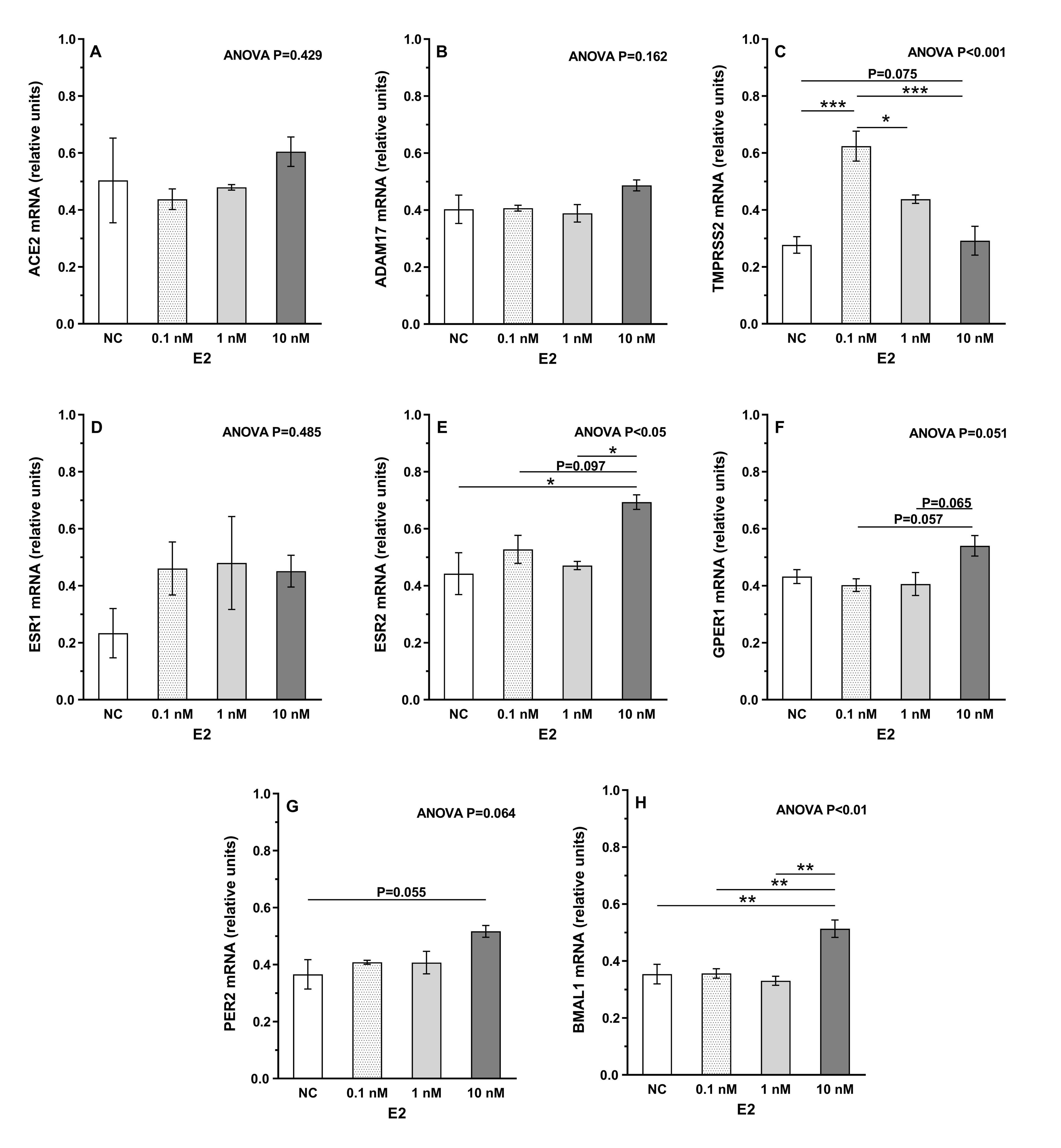

Supplement: S6 Fig — Effect of 17β-estradiol (E2) administration on ACE2 (A), ADAM17 (B), TMPRSS2 (C), ESR1 (D), ESR2 (E), GPER1 (F), PER2 (G) and BMAL1 (H) mRNA expression in DLD1 cells. NC–negative control. * P < 0.05, ** P < 0.01, ANOVA, post hoc Tukey test. (TIF) [file pone.0270609.s006.tif]

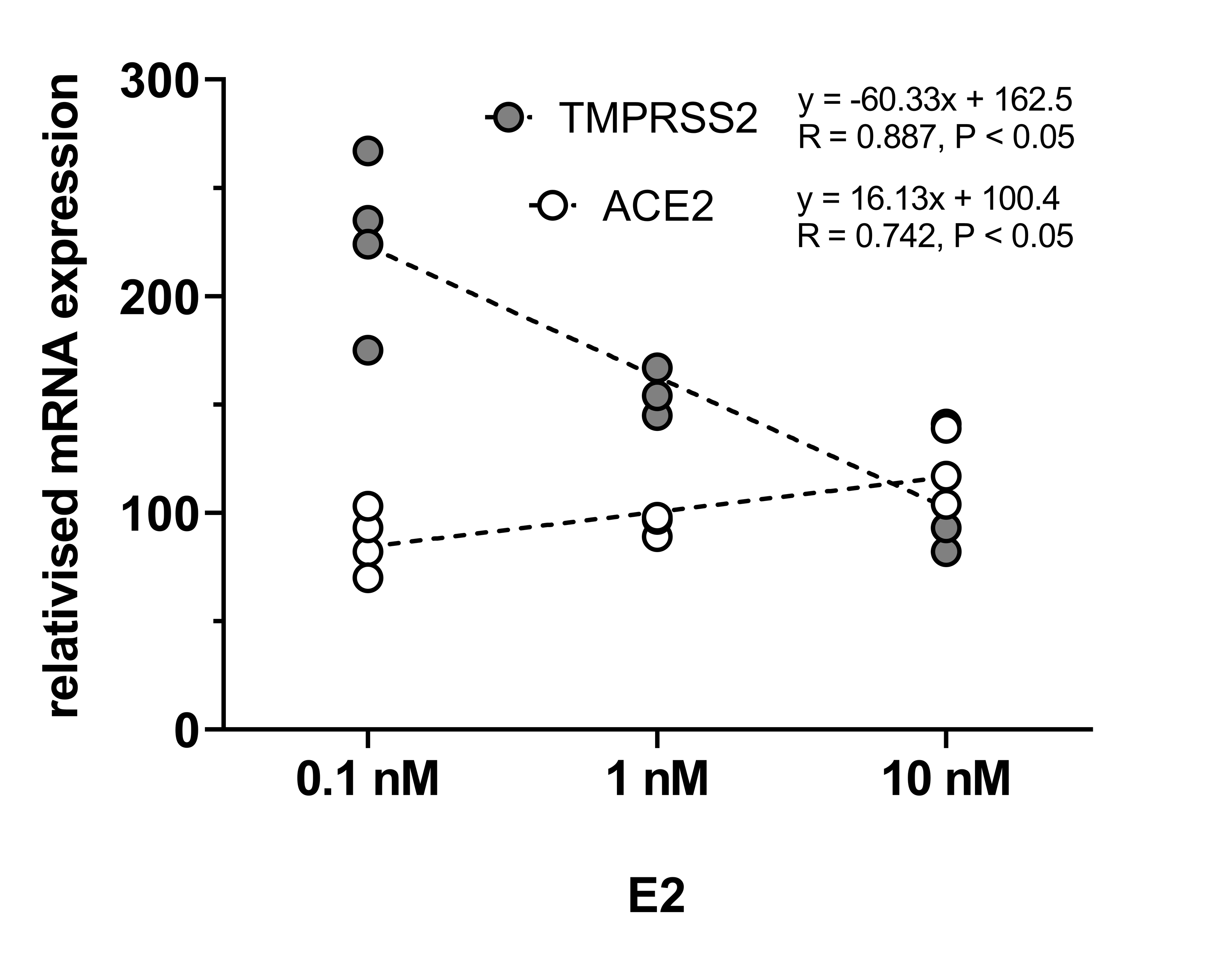

Supplement: S7 Fig — Broken line shows significant fit of linear trend in ACE2 and TMPRSS2 mRNA expression. R–regression coefficient. (TIF) [file pone.0270609.s007.tif]
