# Supplementary material for: Effect of 17β-estradiol on the daily pattern of ACE2, ADAM17, TMPRSS2 and estradiol receptor transcription in the lungs and colon of male rats
Source: PLoS One. 2022 Jun 28;17(6):e0270609. doi: 10.1371/journal.pone.0270609 (PMC9239479; doi:10.1371/journal.pone.0270609)
Supplement: S1 Table — Abbreviations: AT, annealing temperature; ace2, angiotensin converting enzyme 2; adam17, ADAM metallopeptidase domain 17; bAct, beta actin; bmal1, brain and muscle arnt-like protein-1; esr1, oestrogen receptor 1; esr2, oestrogen receptor 2; gper1, G protein-coupled receptor-1; per2, period circadian regulator 2; tmprss2, transmembrane protease/serine protease 2; U6, U6 small nuclear RNA. (DOC) [file pone.0270609.s008.doc]

**S1 Table. Sequences of the primers used in real-time polymerase chain reaction.**

|  | **Gene** | **Strand** | **Sequence (5′ → 3′)** | **Accession number** | **AT** |
| --- | --- | --- | --- | --- | --- |
| **rat** | ***ace2*** | sense | GAC CAA AAA GTG GTG GGA GA | NM_001012006.1 | 49°C |
|  |  | antisense | AGT GGG CCA TCA TGT TTA GC |  |  |
|  | ***adam17*** | sense | GCG ACA CAC TTA GAA ACA CTAC | NM_020306.3 | 49°C |
|  |  | antisense | TTC CTC TTT CCC ATC CAC CA |  |  |
|  | ***bmal1*** | sense | GCA CTC ACA CAT GGT TCC AC | NM_024362.2 | 55°C |
|  |  | antisense | CAT TCC GCA AGG TGT CCT AT |  |  |
|  | ***esr1*** | sense | ATG GGG TCT GGT CCT GTG | NM_012689.1 | 55°C |
|  |  | antisense | GTC TTT TCG TAT CCC GCC TTT C |  |  |
|  | ***esr2*** | sense | TCG TTC TGG ACA GGG ATG AG | NM_012754.2 | 49°C |
|  |  | antisense | GCC AAG GGG TAC ATA CTG GA |  |  |
|  | ***gper1*** | sense | TGC CTG AAT CCC CTC ATC T | NM_133573.2 | 55°C |
|  |  | antisense | TGC TCC CCT GTC CGT TTT C |  |  |
|  | ***per2*** | sense | GAG GTT CAG GGA AGT GAG CA | NM_031678.2 | 49°C |
|  |  | antisense | TTG ACA CGC TTG GAC TTC AG |  |  |
|  | ***tmprss2*** | sense | CAG CAA ATG AGG GCG AAC AG | NM_130424.3 | 49°C |
|  |  | antisense | CCA GGG AGC ACA GTC AGA TAA |  |  |
|  | ***U6*** | sense | GCT TCG GCA GCA CAT ATA CTA A | NR_004394.1 | 49°C |
|  |  | antisense | AAA ATA TGG AAC GCT TCA CGA |  |  |
|  | ***bAct*** | sense | AGC CAT GTA CGT AGC CAT CC | NM_031144.3 | 49°C |
|  |  | antisense | GCT GTG GTG GTG AAG CTG TA |  |  |
| **human** | ***ace2*** | sense | GGA CTC TGC CAT TTA CTT AC | NM_001371415.1 | 53°C |
|  |  | antisense | AAC TAT CTC TCG CTT CAT CT |  |  |
|  | ***adam17*** | sense | AGT GCC AGG AGG CGA TT | NM_003183.6 | 53°C |
|  |  | antisense | GCA GGA GTT GTC AGT TTC ATT AC |  |  |
|  | ***bmal1*** | sense | ACT TCC CCT CTA CCT GCT CAA | NM_001297724.1 | 53°C |
|  |  | antisense | TGT CTT CAT CCA GCC CCA TC |  |  |
|  | ***esr1*** | sense | AGC TTC GAT GAT GGG CTT AC | NM_000125.4 | 53°C |
|  |  | antisense | TTT TCC CTG GTT CCT GTC CAA |  |  |
|  | ***esr2*** | sense | TGA GGG GAA ATG CGT AGA AGG | NM_001437.2 | 53°C |
|  |  | antisense | CGT TCA GCA AGT GAG CCA G |  |  |
|  | ***gper1*** | sense | GGT CTC TTC CTC TCT CTA GCC CT | NM_001098201.3 | 53°C |
|  |  | antisense | CTC CTT GGT CAG GCC CAT CAG |  |  |
|  | ***per2*** | sense | AAT GCC GAT ATG TTT GCG GT | NM_022817.1 | 53°C |
|  |  | antisense | GCA TCG CTG AAG GCA TCT CT |  |  |
|  | ***tmprss2*** | sense | AGG TGA AAG CGG GTG TGA GG | NM_001135099.1 | 60°C |
|  |  | antisense | GCT GTG CGG GAT AGG GG |  |  |
|  | ***U6*** | sense | GCT TCG GCA GCA CAT ATA CTA A | NR_004394.1 | 53°C |
|  |  | antisense | AAA ATA TGG AAC GCT TCA CGA |  |  |
|  | ***bAct*** | sense | GAC CCA GAT CAT GTT TGA GA | NM_001101.2 | 53°C |
|  |  | antisense | GAG GGC ATA CCC CTC GTA G |  |  |

Abbreviations: AT, annealing temperature; *ace2*, angiotensin converting enzyme 2*; adam17*, ADAM metallopeptidase domain 17; *bAct,* beta actin*; bmal1*, brain and muscle arnt-like protein-1; *esr1*, oestrogen receptor 1; *esr2*, oestrogen receptor 2; *gper1*, G protein-coupled receptor-1; *per2*, period circadian regulator 2; *tmprss2*, transmembrane protease/serine protease 2; *U6*, U6 small nuclear RNA.
