# Supplementary material for: Effect of 17β-estradiol on the daily pattern of ACE2, ADAM17, TMPRSS2 and estradiol receptor transcription in the lungs and colon of male rats
Source: PLoS One. 2022 Jun 28;17(6):e0270609. doi: 10.1371/journal.pone.0270609 (PMC9239479; doi:10.1371/journal.pone.0270609)
Supplement: S2 Table — To perform Cosinor analysis a cosine curve with a 24-h period was approximated to the time series data of control and E2-exposed rats. Acrophase–time of curve peak from circadian time zero (dark-to-light transition, ZT0), Mesor–average value of fitted curve, Amplitude–value of curve peak relative to the mesor. Mesor and amplitude are given in relative units. Parameters of rhythms were compared when the fitted curve for both groups showed a significant correlation with the experimental data. P–statistical significance of the fitted cosine curve, R–correlation coefficient, ZT–Zeitgeber time. Gray fields indicate significant differences revealed by Cosinor analysis, * implicates the parameter in which two rhythms differed; trends very close to P < 0.05 are written in italics and a smaller font. ↑ - increase, ↓ - decrease. (DOC) [file pone.0270609.s009.doc]

### **S2 Table: Statistical analysis of gene expression and 17β-estradiol levels in plasma.**

| **Tissue** | **Gene** | **Group** | **Acrophase**  **± SEM [hh:mm]** | | | **Mesor**  **± SEM** | | | **Amplitude**  **± SEM** | | | **P** | **R** | **Effect of E2** | |
| --- | --- | --- | --- | --- | --- | --- | --- | --- | --- | --- | --- | --- | --- | --- | --- |
|  |  |  |  |  |  |  |  |  |  |  |  |  |  | **Cosinor analysis** | **Comparison of time points** |
| **Lungs** | **ACE2** | **Control** | *4:15* | *±* | *1:50* | *1.66* | *±* | *0.05* | *0.16* | *±* | *0.08* | *0.1301* | *0.41* | emergence of rhythm |  |
|  |  | **E2** | 0:35 | *±* | 0:53 | 1.61 | *±* | 0.06 | 0.37 | *±* | 0.09 | 0.002 | 0.66 |  |  |
|  | **ADAM17** | **Control** | 1:33 | *±* | 0:46 | 1.61 | *±* | 0.04 | 0.31 | *±* | 0.06 | 0.0004 | 0.71 |  |  |
|  |  | **E2** | 1:32 | *±* | 0:42 | 1.67 | *±* | 0.06 | 0.46 | *±* | 0.09 | 0.0001 | 0.75 |  |  |
|  | **TMPRSS2** | **Control** | 21:06 | *±* | 2:57 | 1.41 | *±* | 0.04 | 0.08 | *±* | 0.06 | 0.4502 | 0.26 | emergence of rhythm | **↑ ZT22** (P<0.05)  ↑ *ZT2 (P=0.089)* |
|  |  | **E2** | 23:16 | *±* | 0:54 | 1.42 | *±* | 0.05 | 0.28 | *±* | 0.07 | 0.0015 | 0.68 |  |  |
|  | **ESR1** | **Control** | 17:00 | *±* | 1:16 | 1.90 | *±* | 0.07 | 0.32 | *±* | 0.10 | 0.0191 | 0.55 | loss of rhythm | ↑ *ZT2 (P=0.080)* |
|  |  | **E2** | 13:29 | *±* | 2:35 | 2.11 | *±* | 0.19 | 0.39 | *±* | 0.27 | 0.373 | 0.29 |  |  |
|  | **ESR2** | **Control** | 3:02 | *±* | 1:41 | 2.39 | *±* | 0.34 | 1.07 | *±* | 0.48 | 0.1003 | 0.43 | emergence of rhythm | **↑ ZT2** (P<0.05) |
|  |  | **E2** | 2:19 | *±* | 1:01 | 2.75 | *±* | 0.28 | 1.48 | *±* | 0.39 | 0.0044 | 0.63 |  |  |
|  | **GPER1** | **Control** | 0:06 | *±* | 0:00 | 1.51 | *±* | 0.05 | 0.12 | *±* | 0.07 | 0.3158 | 0.32 | emergence of rhythm |  |
|  |  | **E2** | 0:06 | *±* | 0:00 | 1.52 | *±* | 0.06 | 0.36 | *±* | 0.09 | 0.003 | 0.64 |  |  |
|  | **PER2** | **Control** | 14:09 | *±* | 0:14 | 1.34 | *±* | 0.05 | 1.15 | *±* | 0.07 | <0.0001 | 0.96 |  | ↓ ZT2 (P=0.076) |
|  |  | **E2** | 14:29 | *±* | 0:15 | 1.30 | *±* | 0.05 | 1.07 | *±* | 0.07 | <0.0001 | 0.95 |  |  |
|  | **BMAL1** | **Control** | 0:30 | *±* | 0:13 | 3.97 | *±* | 0.16 | 3.89 | *±* | 0.23 | <0.0001 | 0.96 | increased amplitude | ↓ *ZT14 (P=0.074)* |
|  |  | **E2** | 0:28 | *±* | 0:15 | 4.29 | *±* | 0.23 | 4.55 | *±* | 0.33 | <0.0001 | 0.95 |  |  |
| **Colon** | **ACE2** | **Control** | 22:11 | *±* | 1:15 | 0.40 | *±* | 0.02 | 0.07 | *±* | 0.02 | 0.0237 | 0.54 | loss of rhythm | **↑ ZT10** (P<0.05), ↑ *ZT18 (P=0.079)*  **↑ control** *vs.* **E2** – **all samples** (P<0.05) |
|  |  | **E2** | 20:56 | *±* | 2:43 | 0.47 | *±* | 0.02 | 0.05 | *±* | 0.03 | 0.3901 | 0.29 |  |  |
|  | **ADAM17** | **Control** | 1:17 | *±* | 0:55 | 0.53 | *±* | 0.01 | 0.10 | *±* | 0.02 | 0.0022 | 0.65 |  |  |
|  |  | **E2** | 3:01 | *±* | 0:51 | 0.53 | *±* | 0.02 | 0.09 | *±* | 0.02 | 0.0011 | 0.69 |  |  |
|  | **TMPRSS2** | **Control** | 10:24 | *±* | 1:21 | 0.73 | *±* | 0.03 | 0.10 | *±* | 0.04 | 0.0408 | 0.50 | loss of rhythm | **↑ ZT2** (P<0.05) |
|  |  | **E2** | 5:36 | *±* | 3:43 | 0.72 | *±* | 0.02 | 0.03 | *±* | 0.03 | 0.576 | 0.22 |  |  |
|  | **ESR1** | **Control** | 0:38 | *±* | 0:59 | 0.53 | *±* | 0.02 | 0.09 | *±* | 0.02 | 0.005 | 0.62 | loss of rhythm | **↑ ZT6, ZT10** (P<0.05)  ↓ *ZT18 (P=0.05)* |
|  |  | **E2** | 6:14 | ± | 1:34 | 0.58 | ± | 0.03 | 0.10 | ± | 0.04 | 0.0609 | 0.47 |  |  |
|  | **ESR2** | **Control** | 2:40 | *±* | 1:47 | 0.62 | ± | 0.05 | 0.14 | *±* | 0.07 | 0.1303 | 0.41 |  |  |
|  |  | **E2** | 0:54 | ± | 1:31 | 0.72 | ± | 0.06 | 0.19 | ± | 0.08 | 0.0744 | 0.46 |  |  |
|  | **GPER1** | **Control** | 11:05 | *±* | 2:41 | 0.59 | *±* | 0.02 | 0.04 | *±* | 0.03 | 0.4069 | 0.28 |  |  |
|  |  | **E2** | 8:51 | ± | 1:37 | 0.56 | ± | 0.02 | 0.06 | ± | 0.03 | 0.0843 | 0.45 |  |  |
|  | **PER2** | **Control** | 17:00 | ± | 0:32 | 2.97 | ± | 0.17 | 1,77 | ± | 0.24 | <0.0001 | 0.84 | phase advance |  |
|  |  | **E2** | 15:54* | ± | 0:23 | 3.06 | ± | 0.14 | 1.88* | ± | 0.19 | <0.0001 | 0.90 |  |  |
|  | **BMAL1** | **Control** | 1:59 | *±* | 0:18 | 0.49 | *±* | 0.02 | 0.39 | *±* | 0.03 | <0.0001 | 0.93 |  | **↑ ZT2** (P<0.05)  ↓ *ZT6 (P=0.08)* |
|  |  | **E2** | 1:47 | *±* | 0:12 | 0.47 | *±* | 0.02 | 0.43 | *±* | 0.02 | <0.0001 | 0.97 |  |  |
| **Plasma** | **E2** | **Control** | 0:18 | ± | 0:21 | 1.48 | ± | 0.11 | 0.41 | ± | 0.15 | 0.0432 | 0.50 | increased mesor and amplitude | **↑ ZT10, ZT14, ZT18, ZT22, ZT26, ZT30**  (P<0.05) |
|  |  | **E2** | 0:57 | ± | 0:20 | 6.16 | ± | 1.07 | 4.56 | ± | 1.50 | 0.0235 | 0.54 |  |  |

### **S2 Table. Statistical analysis of gene expression and 17β-estradiol (E2) levels in plasma.** To perform Cosinor analysis a cosine curve with a 24-h period was approximated to the time series data of control and E2-exposed rats. Acrophase – time of curve peak from circadian time zero (dark-to-light transition, ZT0), Mesor – average value of fitted curve, Amplitude – value of curve peak relative to the mesor. Mesor and amplitude are given in relative units. Parameters of rhythms were compared when the fitted curve for both groups showed a significant correlation with the experimental data. P – statistical significance of the fitted cosine curve, R – correlation coefficient, ZT – Zeitgeber time. Gray fields indicate significant differences revealed by Cosinor analysis, * implicates the parameter in which two rhythms differed; trends very close to P < 0.05 are written in italics and a smaller font*.* ↑ - increase, ↓ - decrease.
